# Current Advances in the Diagnostic Imaging of Atherosclerosis: Insights into the Pathophysiology of Vulnerable Plaque

**DOI:** 10.3390/ijms21082992

**Published:** 2020-04-23

**Authors:** Nataliya V. Mushenkova, Volha I. Summerhill, Dongwei Zhang, Elena B. Romanenko, Andrey V. Grechko, Alexander N. Orekhov

**Affiliations:** 1 Pharmstandard Ventures, 123112 Moscow, Russia; mushenkova@mail.ru; 2 Department of Basic Research, Institute for Atherosclerosis Research, Skolkovo Innovative Center, 121609 Moscow, Russia; 3 Diabetes Research Center, Traditional Chinese Medicine School, Beijing University of Chinese Medicine, Beijing 100029, China; dongwei1006@gmail.com; 4 Department of Molecular Basis of Ontogenesis, Belozersky Institute of Physical and Chemical Biology, Moscow State University, 119234 Moscow, Russia; romanenkoeb@mail.ru; 5 Federal Research and Clinical Center of Intensive Care Medicine and Rehabilitology, 109240 Moscow, Russia; avg-2007@yandex.ru; 6 Laboratory of Angiopathology, Institute of General Pathology and Pathophysiology, 125315 Moscow, Russia; 7 Laboratory of Infection Pathology and Molecular Microecology, Institute of Human Morphology, 117418 Moscow, Russia

**Keywords:** atherosclerosis, vulnerable plaque, invasive imaging, non-invasive imaging

## Abstract

Atherosclerosis is a lipoprotein-driven inflammatory disorder leading to a plaque formation at specific sites of the arterial tree. After decades of slow progression, atherosclerotic plaque rupture and formation of thrombi are the major factors responsible for the development of acute coronary syndromes (ACSs). In this regard, the detection of high-risk (vulnerable) plaques is an ultimate goal in the management of atherosclerosis and cardiovascular diseases (CVDs). Vulnerable plaques have specific morphological features that make their detection possible, hence allowing for identification of high-risk patients and the tailoring of therapy. Plaque ruptures predominantly occur amongst lesions characterized as thin-cap fibroatheromas (TCFA). Plaques without a rupture, such as plaque erosions, are also thrombi-forming lesions on the most frequent pathological intimal thickening or fibroatheromas. Many attempts to comprehensively identify vulnerable plaque constituents with different invasive and non-invasive imaging technologies have been made. In this review, advantages and limitations of invasive and non-invasive imaging modalities currently available for the identification of plaque components and morphologic features associated with plaque vulnerability, as well as their clinical diagnostic and prognostic value, were discussed.

## 1. Introduction

Cardiovascular diseases (CVDs), including coronary artery disease (CAD) and subsequent acute coronary syndromes (ACSs), continue to dominate as the principal cause of morbidity and mortality in industrialized countries. Annually, hundreds of thousands of American people develop new ACSs, such as unstable angina or silent myocardial infarction [1]. In 2016, 17.9 million people died from CVDs, accounting for 31% of total deaths in the world [2].

It is well known that atherosclerosis is the primary cause of a life-threatening CVD. The formation of atherosclerotic lesions occurs at specific arterial localities (branching sites) that are affected the most by the low and oscillatory endothelial shear stress [3]. According to the current understanding, lesion development involves lipid accumulation in the arterial intima, resulting in foam cell formation, a local inflammatory response, and migration and proliferation of several cell types, including macrophages, smooth muscle cells (SMCs), lymphocytes, neutrophils, and dendritic cells that play a pivotal role in its progression. Lipid accumulation is a key event in the formation of the atherosclerotic lesion, and it is determined by different classes of lipoproteins [4]. Atherosclerotic plaque tends to develop early in life [5], progressing with age; however, the progression rate is not completely predictable and varies among individuals. In general, it undergoes a prolonged asymptomatic phase (lasting many years or several decades) until the manifestation of the first clinical symptoms often at the later stages of atherosclerosis. The mechanisms of plaque progression encompass SMC apoptosis, matrix synthesis, angiogenesis, arterial remodeling, fibrous cap rupture, and thrombosis, followed by necrosis and calcification. The most acute cardiovascular events are triggered by the rupture; erosion; or, the least common, calcified nodule, the vulnerable plaque phenotypes, followed by coronary thrombosis. Ruptured lesions are responsible for the majority (73%) of all ACSs [6]. In addition, the underlying mechanism of sudden coronary death from thrombi was found from plaque erosions, in 30%–35% of cases, and rarely from calcified nodules, in 2%–7% of cases [7].

The biological features of two major classes of high-risk (vulnerable) plaques, such as rupture-prone and erosion-prone plaques, were described [8]. Rupture-prone plaques usually have the morphology of thin-cap fibroatheromas (TCFA) that possess some specific traits, including increased plaque burden and the large lipid-rich necrotic core that is covered by a thin fibrous cap (<65 μm) and infiltrated with macrophages and inflammatory cells [9]. Macrophages were considered as an important factor of plaque vulnerability because they participate in the uptake and metabolism of lipoproteins, growth factor secretion, and production of enzymes and toxic metabolites that promote weakening of the fibrous cap and plaque rupture. Inflammation plays a significant role in plaque progression [10]. Inflammation promotes calcification as a healing response to the necrotic plaque. Calcification begins from an aggregation of small hydroxyapatite crystals forming microcalcification (>50 microns in diameter) embedded in the fibrous cap. Calcifications can aggregate into larger bulks, forming spotty pattern of calcifications of 1–3 mm in diameter. Microcalcifications play an important role in atherosclerotic plaque destabilization. The impact of microcalcifications on plaque stability is determined by the accumulation of local mechanical stress generated within the fibrous cap [11]. However, plaque calcification can be implicated in both progression and regression of atherosclerosis. Progressive calcification supports the transition from initial high-risk microcalcifications to the end-stage macrocalcifications responsible for plaque stability, which limit the inflammation and only occasionally result in rupture [12].

Moreover, a recent study showed that TCFA may contain cholesterol crystals that can disrupt the fibrous cap and potentiate inflammation [13]. In fact, TCFA is the precursor lesion of plaque rupture, where fibrous cap thinning or weakening represents a well-known concept of disruption preceding the plaque rupture. Other features of rupture-prone plaque are large plaque volume with expansive positive remodeling mitigating an obstruction of the lumen, intraplaque hemorrhage, adventitial inflammation, and neovascularization [6]. As a result of plaque rupture, the necrotic core containing collagen fibers becomes exposed to the vessel lumen, leading to the formation of thrombus. 

Erosion-prone plaques are more heterogeneous, lacking distinctive morphological features. Some authors described plaque erosion as an acute thrombus that is in immediate contact with the underlying fibroatheroma in the endothelium uncovered area [14]. Moreover, the primary intimal plaque underlying plaque erosion can be enriched with SMCs and proteoglycan matrix with a minimal or absent inflammation [6,14]. It was indicated that the deep intima of the eroded plaque can frequently contain extracellular lipid pools, whereas the presence of necrotic cores was scarce [7]. In addition, plaque erosions become calcified to a minor degree when compared to plaque ruptures [9]. Nevertheless, their developmental mechanism(s) remains unclear. On the basis of the observation that the media in the segments with the absent endothelium was intact and thicker than that in the sites of plaque rupture [15], a speculative suggestion was made that coronary vasospasm might be implicated in the pathophysiology of plaque erosion [9]. Unfortunately, there is still little knowledge about the sequelae of vasospasm in atherosclerotic vessels. In rare cases, the calcified nodule can also be observed, projecting into the lumen through a fibrous cap rupture, and this was proposed as being an independent triggering mechanism of thrombosis [7].

There is a great necessity in the accurate and reliable diagnosis of vulnerable atherosclerotic plaques prior to clinical manifestations that would help the identification of high-risk patients and tailoring therapy. The rupture-prone plaque components are detectable by a variety of imaging technologies, although it remains challenging to discriminate between erosion-prone plaque and a stable plaque by imaging. Pathological characteristics of erosion-prone plaques have a minor contrast on existing imaging tools, representing a great opportunity for innovation. In this review, we discuss the strengths and limitations of currently available invasive and non-invasive imaging modalities that enable in vivo assessment of different aspects attributed to vulnerable plaques, highlighting their clinical and prognostic value.

## 2. Invasive Imaging

### 2.1. Intravascular Ultrasound

Intravascular ultrasound (IVUS), a catheter-based imaging modality, emerged in the 1990s of the last century [16]. Governed by the accurate and deeply penetrating imaging capacity with the backscattered signal processed in real-time into a two-dimensional (2D) video image, grayscale IVUS enabled in vivo assessment of vessel wall dimensions, phenotypic characteristics, distribution, and severity of the atherosclerotic plaque. Moreover, relying on plaque visual appearance and evaluating its echogenicity compared to surrounding adventitia, grayscale IVUS supported plaque classification as (1) soft plaque, (2) fibrous plaque, (3) calcified plaque, and (4) mixed plaques [17]. Later, advances in IVUS processing and, particularly, the analysis of intravascular ultrasound radiofrequency (IVUS-RF) backscatter signal, also known as virtual histology intravascular ultrasound (VH-IVUS), allowed a real-time cross-sectional and longitudinal three-dimensional (3D) visualization of a vessel that broadened the knowledge on the composition and mechanical properties of the vulnerable plaque [18]. Thus, numerous studies indicated that VH-IVUS potentially improved upon greyscale IVUS, enabling the following: (I) the discrimination between the various types of components of atherosclerotic plaque in greater detail that allowed plaque classification into four distinct groups: (1) fibrous plaque, (2) fibrofatty plaque, (3) necrotic core, and (4) dense calcium [17,19]; (II) providing the morphologic assessment of lesion evolution [20]; (III) the assessment of the incidence and distribution of vulnerable plaques [21,22]; (IV) the assessment of systemic and local risk factor effects influencing the progression of atherosclerotic plaque [23,24,25]; (V) monitoring effects of statin therapy [26]; and (VI) the assessment of plaques before and after percutaneous coronary intervention (PCI) [27,28]. In addition, the VH-IVUS technique was extensively validated [29,30].

However, relying on extensive clinical applications during a period of over 25 years, the accumulated evidence raised concerns about IVUS accuracy in reproducible differentiation of elements of plaque composition and its clinical potential as an independent predictor of major acute coronary events (MACE). The large-scale clinical studies exposed significant limitations of IVUS in cardiovascular risk stratification. The natural history Providing Regional Observations to Study Predictors of Events in the Coronary Tree (PROSPECT) study (NCT00180466), the largest study of this kind, using conventional angiography, grayscale IVUS, and VH-IVUS, examined coronary vessels of 697 patients with ACS that had undergone successful and uncomplicated PCI straight after treatment and during a follow-up period [31]. This study reported that IVUS was not able to visualize the entire coronary tree, assessing only 53% of the lesions that caused adverse cardiovascular events during the median 3.4-year follow-up period. Moreover, multivariate analysis showed the low positive predictive value of only 18.2% in detecting vulnerable lesions that caused MACE during follow-up, in which defined variables included plaque burden >70%, minimal luminal area <4 mm^2^, and the presence of TCFA phenotype. Another study of 70 patients with stable angina or an ACS, who underwent PCI and three-vessel VH-IVUS imaging, presented similar results [27]. In this study, the TCFA presence identified by VH-IVUS was the only parameter predicting MACE related to non-culprit lesions. Moreover, the Prediction of the Progression of Coronary Artery Disease and Clinical Outcomes Using Vascular Profiling of Shear Stress and Wall Morphology (PREDICTION) study (NCT01316159) was the only prospective study that investigated the effects of the low endothelial shear stress on the progression of atherosclerotic disease [32]. It included 506 patients admitted with an ACS, who had PCI and three-vessel grayscale IVUS imaging at baseline and at 6–10 months follow-up. The IVUS data at baseline along with angiographic images were analyzed. Positive predictive value of only 41% (identification of lesions that will require revascularization) was attributed to an increased plaque burden and low endothelial shear stress. Nonetheless, this study was characterized by a high number (33%) of excluded patients due to incomplete data.

The insufficiency of IVUS-based modalities may be reliant on some technical constraints, such as operator-dependent parameters and spatial resolution. Gray-scale IVUS cannot accurately discriminate plaque components, because their relationship with the original acoustic signal is distorted during the processes of the scan conversion and can be further modified by display controls, such as brightness and gain [33]. VH-IVUS has an axial resolution ranging between 100 and 200 μm that limits its ability to identify some features associated with the increased plaque vulnerability, including the fibrous cap thickness (identification of TCFAs with lesser thickness than the spatial resolution of the systems), plaque disruption, macrophage infiltration, and thrombus [34]. Moreover, it is not sufficient to visualize microcalcifications that are a good indicator of rupture susceptibility; however, IVUS can identify large dense calcific plaques or spotty calcifications with high sensitivity and specificity [35].

### 2.2. Optical Coherence Tomography

In the attempt to overcome limitations attributed to IVUS, optical coherence tomography (OCT) was developed as a promising intravascular imaging modality that offered new insights into the atherosclerosis microstructure. Relying on the capacity to provide additional and more specific information on plaque composition important for identifying vulnerable plaques that can cause ACS, OCT was considered as a more potent in vivo technology than IVUS [34,36,37,38]. It was demonstrated that OCT can especially discriminate lipid-rich plaques (fibroatheromas) and visualize fibrous cup thickness, the main characteristics of plaque vulnerability, therefore, allowing reliable evaluation of cap disruption and erosion [34,39]. Another study also confirmed the high efficiency of OCT by showing a prominent correlation between the OCT and histology measurements for fibrous cap thickness, lipid core size, and proportion of lipid content [40]. Moreover, measurement by OCT cap thickness was associated with the prevalence of plaque rupture [41]. OCT has also proven to be beneficial for the quantification of macrophage content in fibrous cups, providing valuable information on the level of plaque inflammation that is a prominent feature of vulnerable plaques [42,43], as well as intraplaque neovascularization, an important contributor to plaque growth and instability [44]. OCT was found to be useful for the assessment of developmental processes, including the formation of a thrombus and calcifications that are significant for the atherosclerotic plaque progression [36,37]. Unlike IVUS, OCT can penetrate plaque calcification and characterize it in detail, in terms of thickness, area, and volume. Accordingly, calcifications classified as macrocalcifications, spotty calcifications, and microcalcifications can be detected by OCT imaging [45].

It was suggested that more extensive calcification is associated with stable plaques [46]. In contrast to macrocalcifications, the presence of spotty microcalcifications detected by IVUS or OCT corresponds with plaque instability. Interestingly, the co-localization of macrophages and microcalcifications in the same plaque was associated with a greater degree of plaque vulnerability, as well as with other features of atherosclerosis, such as increased media thickness, as determined by recent OCT studies [47,48]. Because the same patients showed less advanced coronary artery stenosis, the co-localization of macrophages and microcalcifications is suggestive of an early stage of the atherosclerotic process, which may progress into further calcification and inflammation. These observations indicated that OCT could ensure the assessment of both morphological features and atherosclerotic disease activity. The mutual presence of several high-risk OCT plaque features was linked to a higher risk of MACE [49]. In addition, OCT can visualize stent strut apposition against the vessel wall [50] and neointima formation [51]. However, OCT definition of the necrotic core is scarce, as there is a lack of conclusive published studies directly comparing OCT lipid pool-containing plaques with the necrotic core by histology. 

Technical advantages of OCT included higher resolution (10–20 μm vs. 150–200 μm of IVUS), fast data acquisition rate, small inexpensive designs, and the absence of shadowing artefacts that, in case of calcium deposits, can facilitate visualization of the adjacent tissue [36,52]. Moreover, several OCT-based technologies with improved technical capability that enabled the increased imaging frame rates [53], definition of the collagen composition of fibrous caps with the enhanced resolution and additional contrast mechanisms [54,55], and the visualization of smaller vessels [56] were reported. On the basis of the studies mentioned above, a comparison of OCT and IVUS performance in the structural analysis of the coronary atherosclerosis was made, which is presented in Table 1.

Major limitations of OCT are the low penetration depth of 2–3 mm that may impede the estimation of plaque burden [57] and attenuation of OCT optical beam by intraluminal blood preventing clear visualization of the vessel wall. There are several approaches to overcome the blood interference problem: saline flushes, balloon occlusion, and index matching. Moreover, second-generation OCT technology, such as Fourier-domain OCT, allowing visualization of the microstructure of long coronary artery segments in 3D images, was developed [58].

Despite the extensive usage of OCT in the assessment of plaque pathobiology, there is a lack of clinical studies investigating the fact that OCT-identified plaque morphological characteristics can be potential predictors of plaque progression. There was only one small study of 53 patients that examined OCT efficacy in identifying lesions that are likely to progress and cause MACE [59]. Therefore, OCT-based technology fell short of establishing reliable clinical utility as a predictive tool for risk stratification of ACS.

### 2.3. Near-Infrared Spectroscopy

Another catheter-based intravascular imaging method, near-infrared spectroscopy (NIRS), generally defined as the evaluation of the wavelength-dependent interaction of electromagnetic radiation with the subject matter, became available in the past decade. Relying on its ability to rapidly scan arterial wall both circumferentially and longitudinally, it enabled the analysis of various components of lipid-core plaques in coronary arteries in vivo [60]. NIRS accuracy was validated in human coronary autopsy specimens [61]. Moreover, providing a specific chemical measurement of coronary lipid-rich lesions related to the presence of cholesterol esters in lipid cores, NIRS can also generate spectra that distinguish cholesterol from collagen via their unique spectroscopic patterns [60]. For example, a recent study showed that it was effective for the characterization of fibroatheromas [62]. Moreover, the intracoronary NIRS can be valuable in the prediction of long-term cardiovascular outcomes in non-culprit arteries of CAD patients [63].

Despite the fact that using NIRS catheter presented a reliable and quantitative assessment of lipid-core plaques, compared to any other intravascular imaging methods, NIRS, as a stand-alone method, failed to dominate in a larger range of clinical settings due to some significant limitations. First, NIRS can only give specific information on the lipid constituent but does not support the detailed and complete morphological evaluation of the plaque. Second, it does not allow visualization and assessment of the lumen, outer vessel wall dimensions, and plaque burden. Third, it is deficient in image depth resolution permitting the locating of the necrotic core within the plaque and discriminate TCFA from thick cap fibroatheromas. However, lipid core burden assessment in patients can supplement structural information provided by coronary angiography or IVUS for risk stratification and treatment of patients with ACS.

**Figure 1:**
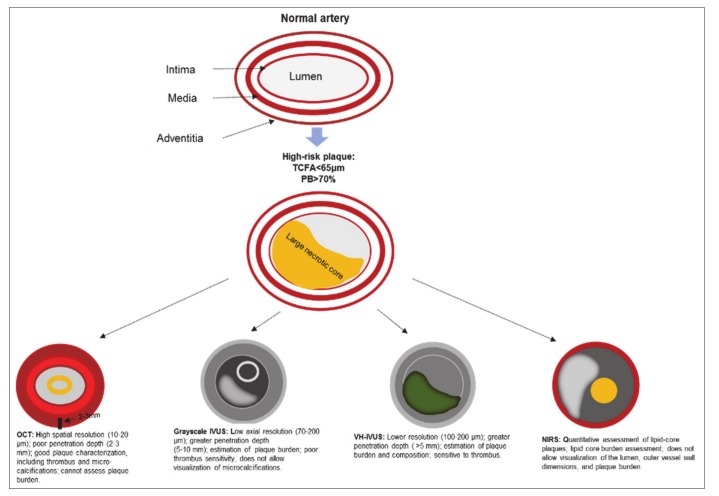
The utility of grayscale IVUS, VH-IVUS, OCT, and NIRS in the visualization of a vulnerable plaque. Note: IVUS—intravascular ultrasound; NIRS—near infrared spectroscopy; OCT—optical coherence tomography; PB—plaque burden; TCFA—thin-cap fibroatheroma; VH-IVUS—virtual histology intravascular ultrasound.

The utility of grayscale IVUS, VH-IVUS, OCT, and NIRS in the visualization of a vulnerable plaque is presented in Figure 1.

### 2.4. Other Intravascular Imaging Technologies

Other intravascular imaging technologies were made available in the preclinical setting, aiming to advance intravascular tissue characterization. Near-infrared fluorescence (NIRF) imaging was suggested for an improved plaque characterization at a cellular and molecular level. It was demonstrated as being able to provide information regarding the activity of plaque inflammation by detection of inflammation-regulated cysteine protease activity in atheroma and coronary stent-induced arterial injury in vivo [64]. A disadvantage to this intravascular approach is the presence of the light attenuating blood that limits the depth resolution of the NIRF signal. Moreover, it was revealed that intravascular photoacoustic imaging (IVPA), the analytical chemistry diagnostic tool, was able to provide information in high detail on plaque chemical composition, in particular, lipids such as cholesterol and cholesterol esters [65,66]. Nonetheless, there are some limitations attributed to IVPA use. Similarly to NIRF, there is a need for a blood-free surface because the blood attenuates IVPA signal [67]. It also has limited ability to visualize the entire plaque for the lipid content evaluation in the presence of large lipid cores.

Furthermore, a flexible fiberoptic-based fluorescence lifetime imaging microscopy (FLIM) technique was developed [68]. Based exclusively on the fluorescence decay dynamics without the requirement for fluorescence intensity information or contrast agents, FLIM can determine biochemical characteristics of plaque fibrotic cap that are directly linked to plaque instability, that is, it can discriminate between lipid pools and collagen-rich regions in fibrous cups that are useful to rate the risk of plaque rupture [69]. Unlike NIRF and IVPA techniques, time-resolved FLIM catheter incorporates a blood flushing system, making its performance unaffected by the presence of blood, and increasing its potential for clinical translation [70].

Overall, different modalities have different capacities for the identification of plaque components, as presented in Table 2.

### 2.5. Multimodality Imaging

To overcome the limitations and augment the reliability of the above-described techniques, intravascular hybrid imaging that combines two different modalities with complementary strengths, allowing more specific and comprehensive evaluation of plaque morphology, pathobiology, and prediction of lesion evolution, was proposed. Thus, the study demonstrated that in providing 3D models, combined IVUS-NIRS imaging can be particularly advantageous in simultaneous identification of the distribution of lipid core plaques and investigation of relations between vessel geometry, shear stress, and plaque composition [71]. Demonstrating more reliable characterization of plaque composition, fused IVUS-NIRS imaging underwent histopathological validation in recent studies [72,73]. For instance, the detection of superficial thinning as the IVUS signature of a fibroatheroma is frequently prevented by the presence of calcification; NIRS can detect lipid regardless of substantial calcification [74]. The improved efficacy of fusion of IVUS and NIRS, compared to their separate use, was also proven by some other IVUS-NIRS studies [74,75]. Because the culprit lesions in patients with non-ST or ST-elevation myocardial infarction (STEMI) have specific morphological characteristics [76], the IVUS-NIRS application was shown to be capable of accurately differentiating STEMI culprit from non-culprit segments [74,75]. In addition, the combined use of IVUS and NIRS was successfully employed to assess the effect of statins on plaque burden and composition [77]. Moreover, the Lipid Rich Plaque study (NCT02033694) indicated that IVUS-NIRS can serve as the first diagnostic tool in the detection of vulnerable plaques and patients at higher risk for subsequent MACE in clinical practice [78]. In addition, there is another prospective clinical study that is currently ongoing, PROSPECT II (NCT02171065), which also investigates the capability of IVUS-NIRS in the identification of patients and plaques vulnerable to MACE. Therefore, considering the great efficacy and utility of IVUS-NIRS, it was the only hybrid intravascular imaging technology approved for clinical use by the Food and Drug Administration in the United States. Nevertheless, limitations of IVUS-NIRS, apart from the above-mentioned loss of the IVUS signal behind calcific tissue, include the low resolution of IVUS that restricts the assessment of cap thickness and lumen border definition in the presence of thrombus or high blood speckle.

Furthermore, the integration of IVUS and OCT, the two most frequently used modalities, combining deep penetration of IVUS and the high resolution of OCT, was suggested. Thus, a dual-modality IVUS-OCT catheter was introduced that was capable of obtaining OCT and IVUS images, simultaneously allowing superior real-time measurements of cap thickness, necrotic core, and plaque burden in both ex vivo and in vitro specimens, as well as in animal models [79,80,81]. Moreover, several human studies reported that the application of combined IVUS and OCT imaging demonstrated their improved imaging properties, providing complementary information for detecting TCFAs [82,83]. In particular, in patients with ACS, the combination of IVUS and OCT techniques can discriminate between plaques of different types, including ruptured culprit plaques responsible for MACE, ruptured non-culprit plaques, and non-ruptured TCFA [41]. Accordingly, it can be used to predict the natural course of plaque progression and further possible clinical complications. In addition, the IVUS-OCT system can be used for coronary stenting imaging, providing information on stent-tissue microstructure [84]. IVUS-OCT human applicability was supported by the prospective cohort study, the Integrated Biomarker Imaging Study (IBIS) 4 (NCT00962416), in which 103 patients treated for STEMI underwent three-vessel IVUS-OCT imaging at baseline and at 13 months follow-up [85]. In this study, the use of combined IVUS-OCT technology was feasible in the majority of patients, and there was no difference in the MACE incidence between patients who had a PCI with and without intravascular imaging during 2 year follow-up period and that confirmed a long-term safety of this method.

Several other hybrid catheters were developed to address certain limitations of IVUS and OCT and broaden the knowledge about morphology and pathobiology of vulnerable plaque. Thus, dual-modality imaging approaches, including OCT-NIRS [86], OCT-near-infrared fluorescence (NIRF) [87], IVUS-NIRF [88], IVUS-intravascular photoacoustic (IVPA) [89], and IVUS-fluorescence life-time imaging (FLIM) [90] are currently undergoing pre-clinical evaluation. The comparative summary of the efficiency of combined intravascular imaging modalities in the characterization of vulnerable plaques is presented in Table 3.

Moreover, the employment of Raman spectroscopy-FLIM bimodal probe demonstrated high chemical specificity in detecting plaque components in human coronary specimens [91]. The advantage of combining these imaging modalities is to complement FLIM, an intravascular surface imaging technique, with high molecular specificity data from Raman, which is able to distinguish between calcifications, cholesterol, or carotenoids. The study also suggested that this bimodal probe can be combined with OCT/IVUS, adding chemical specificity to these morphologic intravascular imaging methods [91]. The recent animal study also indicated potential applications of combined OCT-Raman spectroscopy technique for chemical analysis of plaque lipid depositions, including triglycerides as the major component [92].

Taken together, catheter-based intravascular imaging technologies has shown particularly remarkable progress in recent years, but none of the single intravascular imaging modalities has become a “gold-standard” for the evaluation of plaque vulnerability. Each method has unique characteristics and intrinsic limitations; hence, a synergistic approach fusing two or more modalities appears more favorable. Extensions to triple-modality combinations—for example, IVPA-IVUS-OCT—can be potentially considered. However, multimodal imaging has significant weaknesses: (1) the invasive nature, (2) the increased cost, (3) the inability to visualize the entire coronary tree, and (4) the inability to provide a complete assessment of coronary artery pathophysiology. Further research and histology-based validation studies are required in order to confirm the advantages of the hybrid imaging modalities in the assessment of plaque morphology and composition that would help to accelerate the translation of dual imaging technology into clinical practice.

## 3. Non-Invasive Imaging

To date, several pathological features of atherosclerosis can be visualized and quantified with non-invasive imaging technologies that represent an exciting alternative approach in the assessment of atherosclerotic plaque vulnerability and risk prediction of future cardiovascular events.

### 3.1. Positron Emission Tomography 

Positron emission tomography (PET) is a non-invasive nuclear imaging technique employing intravenous administration of a radio-labelled molecular ligand (or tracer) for detection of cellular activity to evaluate atherosclerosis-relevant biological processes, such as arterial inflammation, hypoxia, neo-angiogenesis, and microcalcification. In this regard, PET can facilitate a better understanding of the pathobiology of atherosclerosis, prediction of the high risk of events associated with plaque rupture, and monitoring of the efficacy of drug treatments. A radio-labelled glucose analogue ^18^F-fluorodeoxyglucose (^18^F-FDG) taken up by metabolically active cells, such as macrophages, is the most commonly used tracer in PET imaging. Numerous human and animal model studies of atherosclerosis showed that ^18^F-FDG is accumulated within the arterial wall in the direct proportion to the degree of cellular glycolysis, accordingly reflecting the plaque macrophage density and the extent of inflammation [93,94,95,96]. Of note, macrophage density is a histological sign of vascular inflammation [97]. ^18^F-FDG uptake was commonly observed in large arteries (carotid, iliac, and aorta) and in the association with cardiovascular risk factors [98]. Vascular FDG-PET signals were shown to be significantly correlated with the presence of traditional cardiovascular risk factors, including obesity, male gender, older age (>65), smoking, hypertension, diabetes mellitus, and hypercholesterolemia [99], being indicative of the prevalence of regional arterial inflammation in patients with these risk factors and significant predictive value of FDG-PET imaging in disease progression. In particular, ^18^F-FDG-PET imaging was considered to be able to predict atheroma progression, adding to the knowledge of how inflammation of atherosclerotic plaque is related to calcification. It was established that focal arterial inflammation precedes subsequent calcification in the same arterial territory, supporting the concept that calcification represents the late stage of atherosclerosis [100]. Moreover, it was shown that aortic inflammation measured by ^18^F-FDG-PET can provide an increasing predictive value of future cardiovascular events beyond traditional risk factors [101]. Arterial FDG uptake may also predict plaque rupture and adverse clinical cardiovascular outcomes [102]. In addition, FDG-PET imaging can serve as a surrogate marker, not only for the atherosclerotic disease activity but also for the efficacy of pharmacotherapies that were confirmed by multiple clinical trials [103,104].

PET’s advantages are related to its excellent sensitivity and quantitative efficiency, permitting detection of picomolar concentrations of a tracer. However, FDG-PET imaging technology is not without limitations. First, its spatial resolution of ≈6 mm is limiting for the direct quantification of the vulnerable plaque in smaller vessels, that is, coronary arteries [105]. Second, the significant cardiac and respiratory motion during coronary imaging procedure can lead to further PET signal degradation, making it difficult to assess the mid to distal coronary tree [105]. PET images are usually fused with computed tomography (CT) or magnetic resonance imaging (MRI) for the accurate anatomical signal localization. The substantial FDG uptake can occur in the adjacent myocardium (particularly under ischemic and/or non-fasting conditions), making it more difficult to distinguish between arterial inflammation and the uptake by myocardial milieu [106]. In this respect, several other PET tracers with greater specificity, including ^68^Ga-DOTATATE [107], ^11^C-PK11195 (targeting receptors of translocator protein) [108], and ^18^F-FMCH (targeting cellular membranes of macrophages) [109] were explored for the detection of arterial inflammation in atherosclerosis. There was lower background myocardial cell uptake with the use of these tracers when compared with ^18^F-FDG, making them preferable for coronary artery imaging.

Development of new PET tracers is an area of intensive research. Within an inflamed plaque, hypoxia, neo-angiogenesis, and microcalcifications also contribute to plaque vulnerability; these processes can be detected with PET using novel tracers, such as ^18^F-FMISO, ^68^Ga-NOTA-RGD, and ^18^F-NaF, respectively [106,110,111]. In particular, ^18^F-NaF tracer has an important advantage over ^18^F-FDG, as the signal is not influenced by myocardial uptake. The ongoing prospective multicenter Prediction of Recurrent Events With 18F-Fluoride (PREFFIR, NCT02278211) study aims to evaluate the prognostic value of coronary18F-NaF PET-CT imaging in patients with myocardial infarction and proven multivessel CAD.

Additional disadvantages of PET include exposure to radiation, relatively bulky reagents of restricted longevity, cost, and limited availability, compared to established modalities.

### 3.2. Computed Tomographic Coronary Angiography

Validation histology and intravascular-based imaging studies showed that computed tomographic coronary angiography (CTCA) is a non-invasive imaging alternative that enables accurate evaluation of the luminal and outer vessel wall dimensions, high-risk plaque burden and morphology, and remodeling pattern [112,113,114,115]. In particular, CTCA allows classification of high-risk plaques as calcified, non-calcified, or partially calcified type (including both calcified and non-calcified plaque tissue). Nevertheless, numerous studies showed that, due to a limited imaging resolution, CTCA has limited accuracy in differentiating between lipid and fibrotic tissue components [115,116]. Relying on histology-based studies, it was established that CTCA can allow characterization of the plaque phenotype and detection of high-risk vulnerable lesions with high specificity but low sensitivity [117,118]. For all this, there is uncertainty as to whether these data can implicate prognostic information and the estimation of the value of CTCA in the prediction of atherosclerosis progression, with prediction of high-risk plaques being a current subject matter of clinical research. The recent systematic review and meta-analysis evaluating the correlation between CTCA-derived plaque characterization and MACE demonstrated that many CTCA-defined plaque characteristics may predict the occurrence of subsequent cardiovascular events [119]. The major disadvantage of this technique is the requirement for radiation exposure and an iodinated contrast agent [120].

It is generally accepted that plaques in coronary bifurcations are exposed to endothelial shear stress that can be linked to the location of plaque rupture. To assess the relationship among plaque composition, endothelial shear stress, and the location of plaque rupture in human coronary arteries, CTCA can be fused with intravascular modalities, such as IVUS and OCT. In a small study, using combined CTCA and IVUS imaging, low endothelial shear stress was associated with thicker plaques, mainly containing IVUS-detected fibrofatty tissue [121]. Additionally, the combination of CTCA and IVUS was used to simulate a stent deployment procedure, demonstrating the potential of in silico methodologies to plan and optimize bifurcation stenting [122]. The fusion of CTCA and OCT was used in segments implanted with bioresorbable vascular scaffolds to investigate the relationship between endothelial shear stress and fibrous cap thickness [123]. This study underscored the significant role of hemodynamics in the modulation of the long-term vascular healing response—the segments exposed to high endothelial shear stress at 2 years post-operation correlated with a thicker fibrous cap at the 5 year follow-up.

### 3.3. Magnetic Resonance Imaging

On the basis of sophisticated imaging advances that accomplish high spatial resolution, black-blood, and minimal motion interference, magnetic resonance imaging (MRI) demonstrated a good ability to provide detailed information on the artery wall morphological parameters, such as wall volume, thickness, and plaque burden, and, moreover, to determine plaque constituents that play an important role in plaque instability [124,125,126]. In particular, non-contrast T1-weighted MRI imaging was shown to be able to identify the presence of thrombus and high-risk plaques [127]. Moreover, MRI can detect positive arterial remodeling in asymptomatic patients with subclinical atherosclerosis [128]. Importantly, MRI was found to be able to visualize the proximal regions of the native coronary arteries [129].

In the atherosclerosis research, MRI has major advantages over CTCA, including better quality evaluation of soft tissue characteristics, lack of the blooming artefacts seen in the calcified plaques, and no exposure to radiation. However, an initial study examining the efficacy of MRI in detecting obstructive CAD, that is, degree of lumen stenosis, which also can serve as a marker of vulnerable plaques, showed moderate accuracy [130]. Subsequent studies that utilized a more advanced imaging technique reported increased accuracy of MRI [131,132,133].

MRI has several limitations to overcome prior to routine clinical application in the atherosclerotic plaque imaging [134]. In comparison to other modalities, MRI scan times can take longer due to a high signal-to-noise ratio, which is required for the high spatial resolution essential for the differentiation of plaque components. In addition, MRI cross-sectional image production may be burdensome, taking up to several minutes, depending on the MRI sequence used and the vessel being investigated. This may pose difficulties in cases of a necessity for a broader coverage or multiple sequences for plaque differentiation. High-resolution MRI for the evaluation of plaque morphology can be limited to a single plaque or vascular segment, making plaque morphology assessment challenging over larger sections of the arterial tree. In addition, exclusive to MRI, patients with claustrophobia and metal devices, such as pacemakers, defibrillators, and certain aneurysm clips, have to be excluded from the MRI procedure.

The advantages and disadvantages of PET, CTCA, and MRI techniques are summarized in Table 4.

### 3.4. Nanotechnology and Molecular Imaging of Atherosclerosis

Nanotechnology is a multidisciplinary research area implicating design, synthesis, and characterization of materials, including nanoparticles and nanostructures with controlled shapes and sizes at the nanoscale (10^−9^ meters) typically ranging from 1 to 100 nm. Nanoparticles of these dimensions exhibit unique physicochemical and biological features, including the ability to cross the cell membrane and tissue barriers, hence permitting the interaction with intracellular structures of similar sizes, such as proteins and other macromolecules with a high degree of reactivity and specificity [141]. However, slightly larger structures also can be considered as nanoparticles [142]. There are several types of nanoparticles, including iron oxides, gold, dendrimers, liposomes, micelles, and biodegradable polymers. In controlled processes, they can stimulate, respond, and interact with target cells or tissues generating desired physiological responses and at the same time minimizing adverse effects. In this way, nanoparticles represent a versatile platform for targeted molecular imaging of different molecules overexpressed in atherosclerotic plaques [143]. By targeting specific molecules, the distribution of contrast agents can be traced precisely to atherosclerotic lesions, and the signal intensity of different imaging modalities can be increased. On that account, molecular imaging provides great potential for non-invasive visualization of the cellular and molecular components engaged in the development of vulnerable atherosclerotic plaques (Table 5).

Hybrid nanoparticles and nanocomposites can produce multifunctional qualities with great potential for more than one clinical purpose, such as theranostic applications [166]. For example, in atherosclerosis, using hybrid lipid-latex nanoparticles can represent an effective strategy for both the selective targeting of plaque M1 macrophages and image-guided therapy, that is, to release a load of therapeutic materials, such as anti-inflammatory drugs [167]. The study also demonstrated improved MRI sensitivity with the use of these hybrid nanoparticles. Successful theranostic applications of other hybrid nanoparticles were also reported [168,169].

There are certain limitations and challenges for plaque-targeted imaging. The problems included nanoparticle surface opsonization, which renders targeting moieties ineffective, nanoparticle uptake by the reticuloendothelial system, and toxicity. Inorganic iron oxide nanoparticles may alter the cytokine profile of macrophages and be toxic in high concentrations [143]. Moreover, due to the low sensitivity of some imaging modalities (MRI), current contrast agents are restricted to the targeting of a few biological molecules with high expression levels in atherosclerotic plaques. Moreover, in plaques, overexpressed molecules vary in different stages of atherosclerosis progress. Therefore, single target imaging cannot provide complete structural, functional, and molecular information of the targeted plaque area. Prolonged retention of targeted tracers also has to be taken into account. In this respect, some dendrimer-based contrast agents, such as polyamidoamine G2 (PAMAM-G2) and polypropylenimine diaminobutyl G3 (DAB-G3) and (DAB-G2) dendrimers showed a relatively rapid excretion that potentially made them acceptable for clinical use [170].

**Figure 2:**
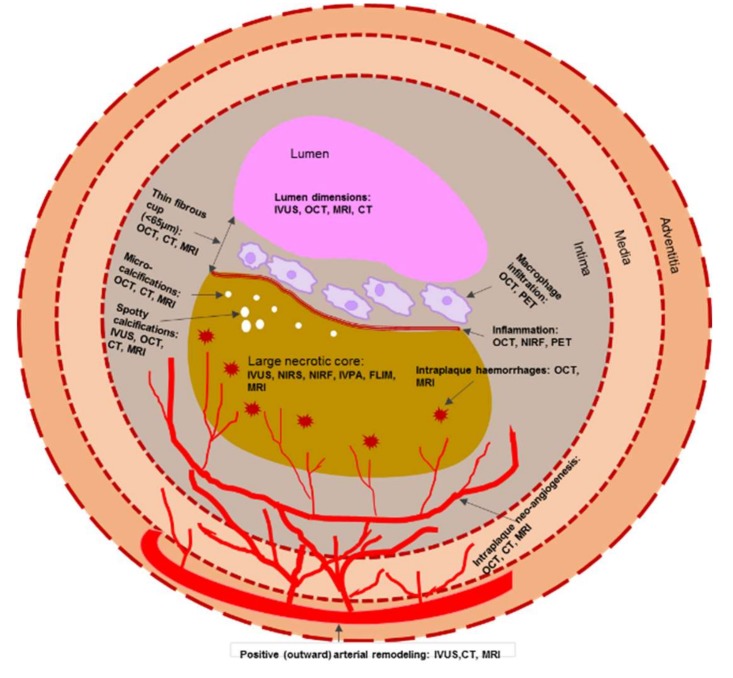
Schematic representation of morphological components of a vulnerable plaque that can be detected both by invasive and non-invasive imaging modalities. Note: CT—computed tomography; FLIM—fluorescence lifetime imaging microscopy; IVPA—intravascular photoacoustic imaging; IVUS—intravascular ultrasound; MRI—magnetic resonance imaging; NIRF—near-infrared fluorescence; NIRS—near infrared spectroscopy; OCT—optical coherence tomography; PET—positron emission tomography.

Taken together, the morphological features of a vulnerable plaque that can be detected both by invasive and non-invasive imaging modalities are presented in Figure 2.

## 4. Identifying Vulnerable Patient

It was accepted that appropriate prevention and treatment strategies require accurate assessment of not only atherosclerotic plaque composition but also cardiovascular risk factors [171]. The identification of traditional cardiovascular risk factors, such as hyperlipidaemia, hypertension, cigarette smoking, diabetes mellitus, and sedentary lifestyle, represents an effective initial step in determining those at risk for MACE. Around 85%–90% of patients with chronic heart disease have one or more of the traditional risk factors [172]. Remarkably, exposure to one or more risk factors is highly predominant in symptom-free individuals, making it difficult to discriminate between low-, intermediate-, and high-risk subjects. Robust evidence emerged, indicating that non-invasive imaging, specifically CT, has the potential to provide additional information to the established risk classification, and that the direct measurements of the subclinical disease may improve identification of a vulnerable patient. For example, multi-slice CT can provide prognostic information, in addition to traditional risk-stratification algorithms, such as the Framingham risk score and coronary artery calcium (CAC) scoring. The Framingham risk score, considering traditional risk factors, is one of the most frequently used to predict the 10 year risk of MACE. The study showed that multi-slice CT imaging was useful in the estimation of subclinical atherosclerosis in different arterial sites, in relation to Framingham Risk Score, and that it can improve patients’ risk stratification [173].

Atherosclerosis is an active process, in which the lesion morphology and composition may undertake a transformation over a few months. Most vulnerable plaques develop from high-risk to more stable and profoundly calcified plaques, whereas others undergo subclinical rupture and healing. Despite the fact that extensive plaque calcification is commonly associated with stable atherosclerosis, there is a robust correlation between the extent of coronary artery calcification and the degree of atherosclerosis and the incidence of future MACE. Extensive calcification is directly related to CAD higher risk. In this respect, several consensus guidelines on the role of CAC scoring in risk stratification of asymptomatic individuals were published [174,175]. Using CTCA, it was found that CAC significantly correlated with atherosclerotic plaque burden, hence providing a direct link to the assessment of MACE risk [176]. Another study also indicated that the extent of plaque detected by CTCA can enhance the risk assessment. Patients with non-obstructive extensive CAD showed similar MACE rates as those with obstructive less extensive CAD [177]. Moreover, the CAC score, as measured by electronic beam CT, can be an independent predictor of coronary heart disease in asymptomatic individuals. A direct association between CAC and the incidence of chronic heart disease was observed in a large cohort of the asymptomatic population of men and women with a wide age range [178]. In symptomatic patients, combining assessment of traditional risk factors with CAC scoring supports a considerable sensitivity for prediction of significant coronary stenosis (over 50%) with higher specificity when assessed by CTCA [179]. Negative CTCA findings correlated with a low risk of MACE [180]. CTCA is mostly suitable for symptomatic moderate-risk patients without known CAD [181]. Therefore, severe CAD can be ruled-out with CTCA, reducing the need for invasive tests. The importance of CAC in the MACE prediction was also demonstrated by the observation that asymptomatic individuals and without conventional risk factors but with high CAC had a markedly higher incidence of all-cause mortality than those who had multiple risk factors but no CAC [182]. In addition, rapid advances in CT imaging provided reduced radiation dose CAC scoring without compromising the imaging quality [183].

These studies clearly indicated that non-invasive CT imaging is appropriate in primary prevention that can improve risk stratification for MACE and identify individuals who may benefit from intensive therapy.

## 5. Conclusions

Despite the fact that none of the imaging modalities described above can provide complete and comprehensive assessment of all signs of plaque vulnerability and mechanisms of atherosclerosis progression, the advances in invasive and non-invasive imaging technology demonstrated their significant diagnostic and prognostic value. A clear understanding of the relationship between vulnerable plaques and vulnerable patients can facilitate not only reliable clinical risk prediction but also a selection of optimal therapy. Large-scale clinical studies are required to demonstrate how current technological advances can be translated from attractive images into imaging strategies that can be widely adopted by clinical settings.

## Figures and Tables

**Table 1:** Comparison of the performance of optical coherence tomography (OCT) and intravascular ultrasound (IVUS) in the structural analysis of coronary atherosclerosis.

Morphological Features	IVUS	OCT
Internal elastic lamina	−	+
External elastic lamina	−	+
Lumen dimensions	+	++
Fibrous plaque	+	++
TCFA < 65 μm	−	+
Necrotic core	+	−
Lipid pools	+	+
Plaque burden	++	+
Microcalcifications	−	+
Spotty calcifications	+	++
Macrocalcifications	+	++
Fibrous cup erosion	−	+
Fibrous cup disruption	+	++
Macrophages	−	+
Thrombus	−	+
Intraplaque neovascularization	−	+
Neointima formation	−	+

**Note:** (−) modality is unable to provide information of this type; (+) modality is capable to provide information of this type; (++) modality has superior ability.

**Table 2:** The capacity of intravascular modalities in the assessment of vulnerable plaque components.

Characteristics of Vulnerable Plaque	Imaging Modality
Fibrous cup thickness (TCFA < 65 μm)	OCT
Necrotic core	IVUS, NIRS, NIRF, IVPA, FLIM
Inflammation	OCT, NIRF
Positive arterial remodeling	IVUS
“Spotty” and microcalcifications	IVUS, OCT
Neo-angiogenesis	OCT
Fibrous cup disruption, erosion, and thrombus	OCT

**Note:** FLIM—fluorescence lifetime imaging microscopy; IVPA—intravascular photoacoustic imaging; NIRF—near-infrared fluorescence; NIRS—near infrared spectroscopy; OCT—optical coherence tomography; VH-IVUS—virtual histology intravascular ultrasound.

**Table 3:** Comparison of the performance of combined intravascular imaging modalities in the characterization of vulnerable plaques.

Characteristics of Vulnerable Plaque	Combined Imaging Modality						
	IVUS-OCT	IVUS- NIRS	OCT-NIRS	IVUS-NIRF	OCT-NIRF	IVUS-IVPA	IVUS-FLIM
Lumen dimensions	***	***	***	***	***	***	***
Plaque burden	***	***	*	***	*	***	***
Positive arterial remodelling	***	***	*	***	*	***	***
Lipid pool	**	***	***	*	**	**	**
Fibrous cap thickness	***	**	***	*	***	*	***
Neo-angiogenesis	**	NA	**	NA	**	*	*
Inflammation	*	NA	*	***	***	**	**

**Note:** (***)—excellent modality performance; (**)—moderate modality performance; (*)—modest modality performance; (NA)—non-applicable (the modality is unable to provide information of this type).

**Table 4:** The summary of the advantages and disadvantages of non-invasive imaging modalities.

Imaging Modality	Advantages	Disadvantages	References
**PET**	Established molecular imaging modality for identification and quantification of inflammation of atherosclerotic plaques and prediction of the natural course of atherosclerosis and risk of MACE. High reproducibility over the short term. High sensitivity. Monitoring of the effectiveness of therapeutic substances.	Not available for wide use. Requires radiotracer. Challenging for imaging of coronary arteries. Expensive.	[95,103,104,105,135]
**CTCA**	Established molecular imaging modality with high specificity and good predictive value.	Low sensitivity: low spatial resolution causing difficulty in distinguishing between lipid rich and fibrous type plaques. Requires radiation exposure and an iodinated contrast agent.	[117,119,120,136,137,138]
**MRI**	Has a good ability to provide detailed information on the artery wall morphological parameters, luminal area, and plaque composition. Suitable for serial studies. Safe, no ionizing radiation. Suitable molecular imaging.	Long scan time. Not suitable for patients with metal devices.	[124,126,127,128,134,139,140]

**Note:** CTCA—computed tomographic coronary angiography; MRI—magnetic resonance imaging; PET—positron emission tomography.

**Table 5:** Targeting of cellular and molecular components of vulnerable atherosclerotic plaques with molecular imaging.

Molecular Target	Plaque Component/Feature	Nanoparticle/Molecular Probe	Imaging Technique	References
VCAM-1 P-selectin	macrophage content ECs	DT-MPIO ^18^F-4V 99mTc-B2702p1	MRI PET–CT SPECT	[144,145,146]
α_v_β_3_-integrin	angiogenesis	Gd-DTPA-BOA fumagillin IONP	MRI	[147,148,149]
OSEs	oxLDL-enriched macrophages	G8 dendrimers modified by manganese and antibody MDA2 manganese micelles LUSPIOs	MRI	[150,151,152]
p32 proteins	activated macrophages	(LyP-1)_4_-dendrimer-^64^Cu	PET–CT	[153]
Au-HDL	macrophage burden, calcification, and stenosis	Au-HDL	Spectral CT	[154]
Macrophage scavenger receptor (CD204)	macrophage content	Gd-carrying immunomicelles	MRI	[140]
Macrophage membrane receptor (CD163)	CD163-expressing macrophages	NP-CD163(m)	MRI	[139]
CD68	macrophages	CD68-Fe-HSNs	US–MRI	[155]
LOX-1	macrophages, SMCs, apoptosis, MMP-9	^111^In-liposome—LOX-1 Ab-DiI Gd-liposome—LOX-1 Ab-DiI	SPECT– CT MRI	[156]
CD44	CD44-expressing macrophages	HA-GdIO NPs	T _1_–T _2_ dual-model MRI	[157]
SR-A	activated macrophages	Fe-PFH-PLGA/CS-DS NPs	MRI, LIFU	[158]
CD80	macrophages, DCs	carbon-11 [^18^F]FDM [^18^F]FDG	PET	[159,160]
MMPs	activity of MMPs	RP805 CGS 27023A	micro-SPECT scintigraphy	[161,162]
Elastin	vascular remodeling	Gd-based elastin specific contrast agent (LMI1174)	MRI	[163]
CB2 receptor	macrophages	[^11^C]RS-016	PET	[164]
NGAL	activity of MMP-9	NGAL/24p3 micelles	MRI	[165]

**Note:** Au-HDL, gold high-density lipoprotein nanoparticle; α_v_β_3_-integrin, alpha(v)beta(3)integrin; CD68-Fe-HSNs, biodegradable Fe-doped hollow silica nanospheres conjugated with anti-CD68 antibody; CGS 27023A, radioligand; [^11^C]RS-016, cannabinoid receptor type 2 (CB2)-specific radiotracer; CT, computed tomography; DC, dendritic cells; DT-MPIO, dual-targeted microparticles of iron oxide; ECs, endothelial cells; ^18^F-4V, ^18^F-labeled tetrameric peptide-PET imaging reporter targeted to VCAM-1; Fe-PFH-PLGA/CS-DS NPs, Fe-PFH (phase transitional material perfluorohexane)-poly(lactic-*co*-glycolic acid) (PLGA)/chitosan (CS)-dextran sulfate (DS) nanoparticles; [^18^F]FDM, 2-deoxy-2-[^18^F] fluoro-D-mannose; [^18^F]FDG, 2-deoxy-2-[^18^F] fluoro-D-glucose; Gd-DTPA-BOA, gadolinium–diethylenetriamine pentaacetic acid-bis-olcate; Gd, gadolinium; HA-GdIO NPs, gadolinium-doped oxide nanoparticles functionalized by hyaluronic acid; IONP, iron oxide nanoparticle; LIFU, low-intensity focused ultrasound; LOX-1, lectin-like oxidized low-density lipoprotein receptor-1; LUSPIOs, lipid-coated ultra-small superparamagnetic iron particles; MMP, matrix metalloproteinase; LyP-1, a cyclic 9-amino acid peptide; MRI, magnetic resonance imaging; NGAL, neutrophil gelatinase-associated lipocalin; NP-CD163(m), gold-coated iron oxide nanoparticles vectorized with an anti-CD163 antibody; OSEs, oxidation-specific epitopes; oxLDL, oxidized low-density lipoprotein; PET, positron emission tomography; RP805, ^99m^Tc-labeled MMP-targeted tracer; SPECT, single-photon emission computed tomography; US-MRI, ultrasound-magnetic resonance imaging dual-modality; VCAM-1, vascular cell adhesion molecule-1; VEGFR, vascular endothelial growth factor receptor; 99mTc-B2702p1, radiotracer.
